# Oocyte-specific Wee1-like protein kinase 2 is dispensable for fertility in mice

**DOI:** 10.1371/journal.pone.0289083

**Published:** 2023-08-01

**Authors:** Kaori Nozawa, Zian Liao, Yuhkoh Satouh, Ting Geng, Masahito Ikawa, Diana Monsivais, Martin M. Matzuk

**Affiliations:** 1 Center for Drug Discovery, Baylor College of Medicine, Houston, TX, United States of America; 2 Department of Pathology & Immunology, Baylor College of Medicine, Houston, TX, United States of America; 3 Department of Experimental Genome Research, Research Institute for Microbial Diseases, Osaka University, Suita, Osaka, Japan; 4 Institute for Molecular and Cellular Regulation, Gunma University, Maebashi, Gunma, Japan; 5 The Institute of Medical Science, The University of Tokyo, Minato-ku, Tokyo, Japan; 6 Department of Molecular and Cellular Biology, Baylor College of Medicine, Houston, TX, United States of America; 7 Department of Biochemistry and Molecular Pharmacology, Baylor College of Medicine, Houston, TX, United States of America; University of Massachusetts Amherst, UNITED STATES

## Abstract

Wee1-like protein kinase 2 (WEE2) is an oocyte-specific protein tyrosine kinase involved in the regulation of oocyte meiotic arrest in humans. As such, it has been proposed as a candidate for non-hormonal female contraception although pre-clinical models have not been reported. Therefore, we developed two novel knockout mouse models using CRISPR/Cas9 to test loss-of-function of *Wee2* on female fertility. A frameshift mutation at the *Wee2* translation start codon in exon 2 had no effect on litter size, litter production, or the ability of oocytes to maintain prophase I arrest. Because of the lack of a reproductive phenotype, we additionally generated a *Wee2* allele with a large deletion by removing all coding exons. While there was no difference in the total number of litters produced, homozygous *Wee2* female knockout mice with the larger deletion produced fewer pups than heterozygous littermates. Furthermore, there was no difference for key reproductive parameters measured in the mouse models, including ovarian weight, number of ovulated oocytes, or oocytes that underwent *in vitro* maturation. Therefore, as loss of *Wee2* in mice shows only minor effects on overall fecundity, contraceptive development with WEE2 should consider exploiting alternative properties such as gain-of-function or protein-protein interactions, as *Wee2* loss-of-function is likely complicated by biological redundancies with other proteins co-expressed in oocytes.

## Introduction

In oocytes, maintenance of meiotic arrest during folliculogenesis and timing of meiotic resumption during ovulation are essential for female fertility [1,2]. Mammalian oocytes experience two extended periods of meiotic arrest. The first arrest is established perinatally when oocytes reach the dictyate stage of prophase I and enclose into primordial follicles. This arrest is maintained even after primordial follicles are recruited into the growing follicle pool. Then, in preovulatory follicles, the midcycle surge of luteinizing hormone (LH) triggers ovulation, meiotic resumption, and progression through metaphase I (MI), but the oocyte then undergoes a second arrest at metaphase II. Wee1-like protein kinase 2 (WEE2) has been reported as a key regulatory factor in both stages [3,4]. In fully grown, germinal vesicle (GV) stage oocytes, WEE2 is thought to actively suppress the maturation phase promoting factor (MPF) by phosphorylating cyclin-dependent kinase 1 (CDK1; also called CDC2), thereby maintaining prophase I [3,5]. After the LH surge, WEE2 undergoes dephosphorylation, allowing the phosphatase CDC25 to translocate to the nucleus where it dephosphorylates CDK1 thus activating MPF [6]. At MII, entry of a spermatozoa triggers calcium (Ca^2+^) oscillation, activating a downstream calcium/calmodulin-dependent protein kinase (CAMKII) [2]. Phosphorylation-mediated activation of WEE2 by CAMKII leads to inhibition of MPF activity and transition of the oocyte from MII arrest to anaphase [4]. Overall, WEE2 is considered to control both phases of meiotic cycle arrest via regulating the phosphorylation status of MPF.

The WEE kinase family consists of three kinases: MYT1, WEE1, and WEE2, which are conserved in human and mice [7,8]. WEE1 and WEE2 have highly conserved amino acid sequences and both are reported to have an inhibitory role in the cell cycle [9]. MYT1, also known as membrane-associated tyrosine and threonine-specific CDC2-inhibitory kinase (PKMYT1), controls CDK1 phosphorylation and inactivation. WEE2 is expressed only in oocytes and zygotes, while WEE1 and MYT1 are also expressed in somatic cells [4]. *WEE2* mutations were recently reported among infertile patients who demonstrated normal menarche and showed regular menses but displayed impaired fertilization after intracytoplasmic sperm injection (ICSI). Specifically, patients diagnosed with fertilization failure had homozygous missense or frameshift mutations of the *WEE2* gene [10]. The fertilization defects were rescued in the oocytes of one patient by microinjecting *WEE2* cRNA into her oocytes. Considering these reports, WEE2 has been proposed as a kinase target for non-hormonal female contraception by inhibiting oocyte meiosis and also as a treatment for female infertility [11].

Despite the compelling human WEE2 data, surprisingly there are no published *Wee2* knockout (KO) mouse models to serve as pre-clinical models and for analyzing the *in vivo* roles of WEE2. Therefore, we used the CRISPR/Cas9 system to develop two different lines of *Wee2* KO mice to determine its suitability as a female contraceptive target. The phenotype analysis of these two lines demonstrates that loss of WEE2 in mice has minimal effects on female fecundity. Thus, additional studies in other species, including non-human primates, may be needed to fully validate WEE2 as a contraceptive candidate along with investigation of additional WEE2 cell cycle-related proteins.

## Materials & methods

### Ethics statement

Mice were maintained in accordance with NIH guidelines, and all animal procedures were performed with prior approval by the Institutional Animal Care and Use Committee (IACUC) at Baylor College of Medicine and Osaka University.

### Animals, analgesia, and surgical procedures

B6D2F1 was purchased from Japan SLC (Hamamatsu, Shizuoka, Japan), CLEA Tokyo B6D2F1, or Charles River. In-house F1 hybrid mice (C57BL/6J × 129S5/SvEvBrd) were mated with *Wee2* heterozygous mice to expand the line and to generate the *Wee2* heterozygous and *Wee2* homozygous (knockout) mice used in all the studies. For phenotypic analysis, sexually mature female mice were used. Mice were euthanized following an IACUC-approved method of cervical disarticulation with a secondary method of euthanasia performed while the mouse was under isoflurane-induced anesthesia. Surgical procedures were performed following IACUC-approved methods that included the administration of analgesia (slow release 1 mg/kg buprenorphine and slow release 2 mg/kg meloxicam, ZooPharm) one hour prior to surgery. Embryo transfer surgery was performed while the mice were under anesthesia with 1–3% isoflurane mixed with oxygen in a vaporizer that was delivered via a nosecone. Mice were observed after surgery to ensure that they were alert and mobile, and were monitored daily for up to 5 days, with analgesia administration (1 mg/kg buprenorphine and slow release 2 mg/kg meloxicam every 72 hours) to manage pain. Pain or discomfort was assessed using the grimace scale and by monitoring mouse activity. All mice were housed with a 12 h light cycle.

### Generation of *Wee2* knockout mice

Zygotes were harvested from superovulated B6D2F1 females mated with B6D2F1 males or obtained through *in vitro* fertilization using B6D2F1 males and females. For the frameshift mutation, the pX330 plasmids expressing *hCas9* and single guide (sg)RNAs (GACTGCACAAGACATCGGAG) were injected into the pronuclei of zygotes [12]. For the large deletion mutation, the ribonucleoprotein (RNP) complex consisting of the custom sgRNAs (GACTGCACAAGACATCGGAG and AGAAGTGAGTTCCTACACGG, Sigma) and Cas9 protein (Thermo Fisher Scientific) were electroporated into zygotes using an ECM 830 electroporation system (BTX, Holliston MA). Eggs were cultured in KSOM overnight to 2-cell stage embryos and then transferred into the oviducts of pseudopregnant ICR females (purchased from the Center for Comparative Medicine, Baylor College of Medicine). Screening of *Wee2* mutant pups was performed by direct sequencing following polymerase chain reaction (PCR) using primers. Founder mice with the 17 bp or 21878 bp deletions were used to expand the colony. For 17 bp deletion, PCR using the primers (Fw1: 5’-ATGCTGAGGCCTTAGAAGTGTGG and Rv1: 5’-GTGGAGAAGTTGGTAGGGACTGG) was conducted and analyzed by direct sequencing. For 21878 bp deletion, the genotyping was carried out by PCR with specific primers for the WT alleles (Fw1: 5’-ATGCTGAGGCCTTAGAAGTGTGG and Rv1: 5’- GTGGAGAAGTTGGTAGGGACTGG) or KO allele (Fw1: 5’ATGCTGAGGCCTTAGAAGTGTGG and Rv2: 5’-GATACACAAGACCGTTGCTAAGG).

### Reverse Transcription-PCR (RT-PCR) and quantitative RT-PCR (qRT-PCR)

RNA was generated from freshly harvested mouse ovaries using RNeasy Mini Kit (QIAGEN #74104) following the manufacturer’s protocol. For each genotype, 6 ovaries from 3 mice were collected. 500 ng RNA per sample was used for reverse transcription using iScript reverse transcription supermix (BIO-RAD #1708840) in a 20 μl reaction. For RT-PCR, 50 ng of the reverse-transcribed cDNA was used for PCR with specific primer for *Wee2* transcripts (Fw2: 5’-GAGCAGAGTCTTTACCCATCAAT and Rv2: 5’-GATACACAAGACCGTTGCTAAGG) and *Gapdh* transcripts (Gapdh-Fw1: 5’-AGCCTCGTCCCGTAGACAA and Gapdh-Rv1: 5’-AATCTCCACTTTGCCACTGC). For qRT-PCR, SYBR Green Master mix (ThermoFisher #A25742) was used following the manufacturer’s recommendations. *Gapdh* was used as the internal control. All reactions were run in triplicates with each value marked with group symbols as indicated in the figure legends. Primers used were as follows: Gapdh-Fw2: 5’-CAATGTGTCCGTCGTGGATCT, Gapdh-Rv2: 5’ GCCTGCTTCACCACCTTCTT; Wee1-Fw: 5’-CCATTGGCTGGCTCTGTTGATG, Wee1-Rv: 5’-CAGGCAGAGAAATAGCGAACGAC; Myt1-Fw: 5’-GGTCTCACCATCTTGGAAGTGG, Myt1-Rv: 5’-CAGCATCATGGCGAGGACAGAA. Statistical significance was examined by one-way ANOVA with Tukey’s multiple comparisons.

### Female fertility assessment

Sexually mature female mice were continuously pair-housed with wild type F1 C57BL6J/129SvEv hybrid males for two (line *Wee2*^*-17*^) or six (*Wee2*^*-21878*^) months. The number of pups born per litter and the number of litters per female were counted. Average litter sizes are presented as the average number of pups per litter from all the females. For ovulated oocyte counting, *Wee2* HET or KO females were injected with pregnant mare serum gonadotropin (PMSG) (7.5 IU) and human chorionic gonadotropin (hCG) (5 IU) at 48 hours interval. Cumulus oocyte complexes (COC) were harvested from the oviduct ampulla 17 hours after hCG injections and transferred into potassium-supplemented simplex optimized medium (KSOM) medium containing 0.1% hyaluronidase (Type IV-S, Sigma-Aldrich) to remove cumulus cells. Then, the number of oocytes was counted.

### Histology

Ovaries were collected and weighted. Ovaries were subsequently fixed in 10% formalin (Sigma Aldrich) at room temperature overnight followed by incubation in 70% ethanol until further processing. The tissues were processed and embedded in paraffin (the Clinical Pathology Laboratory at Baylor College of Medicine). Five μm sectioned tissues were stained by Periodic Acid-Schiff (PAS)-hematoxylin stain and histology assessed.

### *In vitro* oocyte maturation of *Wee2* HET and *Wee2* KO mice

Fully grown GV oocytes were collected from ovaries of sexually mature *Wee2*^+/-17^ HET, *Wee2*^-17/-17^ KO, *Wee2*^+/-21878^ HET and *Wee2*^-21878/-21878^ KO females 46–47 h after PMSG injection as previously described [13,14]. Antral follicles were punctured with 26G needles in FHM or KSOM with 100 μM dibutyryl-cyclic AMP (dbcAMP) (Sigma). Cumulus cells were dissociated by pipetting through a small bore pipette and washing of the oocytes was performed in minimal essential medium alpha (GIBCO) containing BSA (Sigma) or in KSOM containing 100μM dbcAMP. To test maintenance of meiotic arrest, oocytes from both genotypes were cultured in KSOM medium with dbcAMP for 24 hours. After 24 hours of oocyte culture, dbcAMP was removed from the medium and the number of oocytes that underwent germinal vesicle breakdown (GVBD) were counted.

### Statistical analysis

Statistical significance was evaluated using the two-tailed unpaired Student t-test assuming unequal variances except as otherwise noted. Data are represented as means ± SEM. **** P<0.0001. *** P<0.001. *P < 0.05. ns, not significant. In vitro maturation was analyzed using a chi-square test in GraphPad Prism v9. Non-linear regression analysis in Fig 2E was performed using GraphPad Prism, using equation Y = YIntercept + Slope*X.

## Results

**Fig 1:**
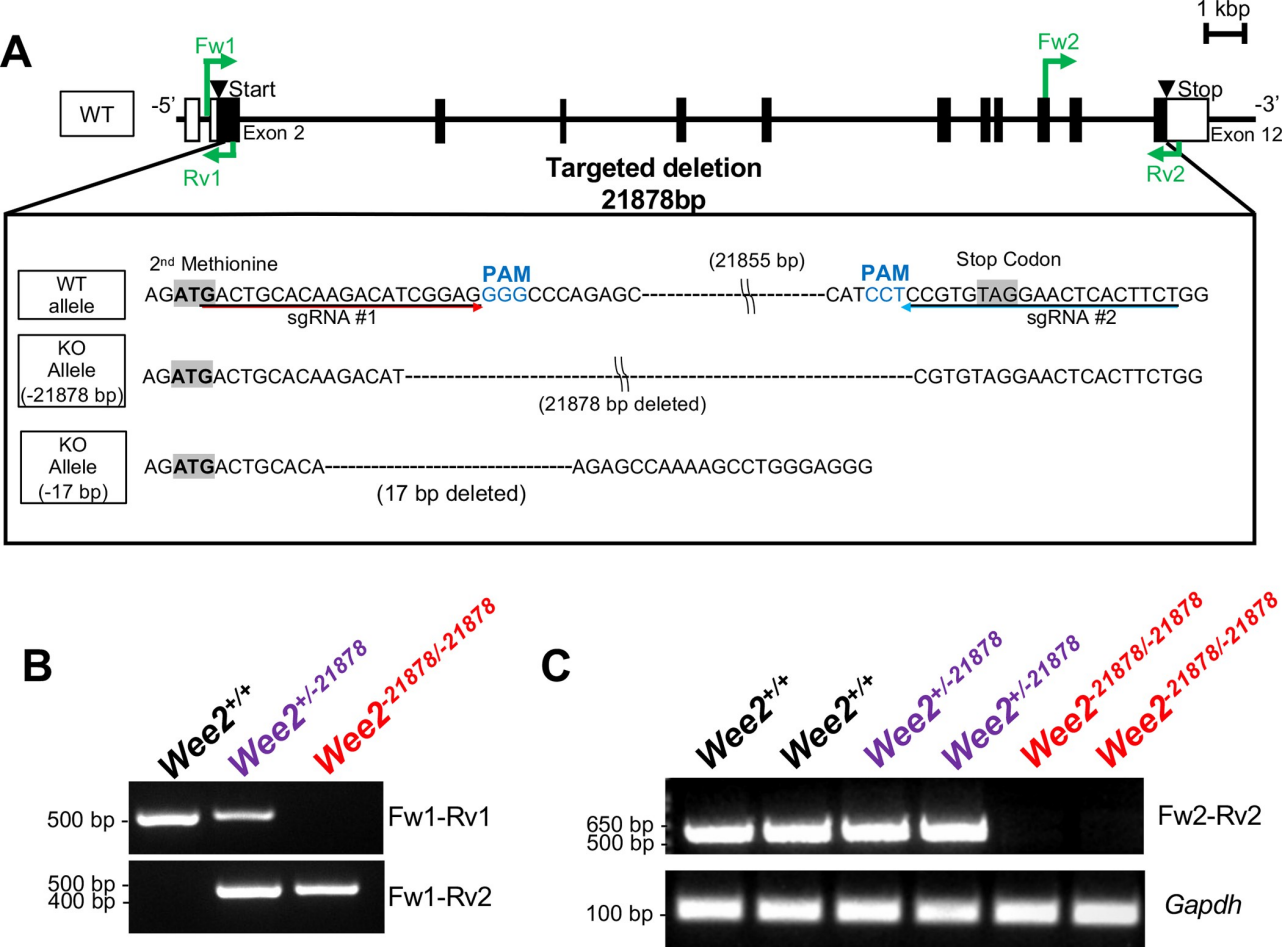
Using CRISPR/Cas9 to generate two lines of *Wee2* KO mice. (A) Genomic structure of mouse *Wee2* and scheme to generate the gene KO mice using the CRISPR/Cas9 system. White and black boxes indicate untranslated and coding regions, respectively. Black under bars indicates sgRNA that targets the region. The delivery of the CRISPR/Cas9 system into zygotes for mutagenesis via electroporation or microinjection resulted in -21878 bp large deletion and -17 bp frameshift deletion, respectively. PAM: Protospacer adjacent motif. (B) Genotyping of *Wee2* alleles for *Wee2*^*-21878/-21878*^ line. Primers positions as indicated in Fig 1A amplify the specific amplicon for the wildtype or mutant allele. (C) RT-PCR of total ovarian RNA in *Wee2*^*-21878/-21878*^ line using primers spanning the region encoding the kinase domain. Expected amplicon sizes for *Wee2* Fw2-Rv2 and *Gapdh* are 569 bp and 104 bp, respectively.

To examine if WEE2 is essential *in vivo*, we generated novel *Wee2* mutant alleles in mice using the CRISPR/Cas9 system. First, we designed sgRNAs to target the region near the codon encoding the 2^nd^ methionine in exon 2 of *Wee2* (Fig 1A). The Cas9 protein and sgRNA were introduced into the cell by injecting 5 ng of pX330 plasmids into the pronuclei of fertilized eggs and culturing them *in vitro* to the 2-cell stage. The 2-cell stage embryos were then transferred into the oviducts of pseudopregnant females and direct Sanger sequencing of the resulting pups identified the presence of a -17 bp deletion (Fig 1A). One founder was selected and intercrossed to obtain subsequent generations. The homozygous -17 bp deletion (*Wee2*^*-*17/-17^) KO mice revealed no obvious developmental abnormalities. To test overall fecundity, at 8 weeks of age, sexually mature *Wee2*^*+/-17*^ (HET) or *Wee2*^*-17/-17*^ (KO) mutant females were pair-housed with a wild type (WT) male for 2 months to test their fertility. Following two months of mating, we found no reduction in litter size between *Wee2*^*+/-17*^ HET females (6.9 ± 1.2 pups/female, n = 3) and *Wee2*^*-17/-17*^ females (4.4 ± 0.91 pups/female, n = 8) (S1A Fig). We observed normal mating behavior in *Wee2*^+/-17^ HET and *Wee2*^-17/-17^ KO females as determined by the presence of copulation plugs indicating that both the *Wee2*^+/-17^ HET and *Wee2*^-17/-17^ KO mice retained the capacity to produce live pups.

Because the frameshift mutation in the *Wee2*^*-17/-17*^ KO mice might result in potential expression of a truncated WEE2 protein with residual activity, we generated another line of knockout mice targeting the second methionine in exon 2 and the stop codon in exon 12, which covers most of the open reading frame (Fig 1A). One-cell stage embryos were electroporated with Cas9 protein and the two sgRNAs. The resulting pups were genotyped and the new CRISPR/Cas9 experiment resulted in a deletion of 21878 bp of *Wee2* and presumably the entire WEE2 kinase domain (Fig 1A and 1B). Realtime PCR (RT-PCR) and quantitative RT-PCR (qRT-PCR) validated the genomic deletion of *Wee2* leads to the absence of *Wee2* mRNA transcripts (Fig 1C; see also Fig 5 below). Thus, the *Wee2*^-*21878*^ deletion allele is a null allele, lacking the ability to encode a potential protein with any WEE2 kinase activity.

**Fig 2:**
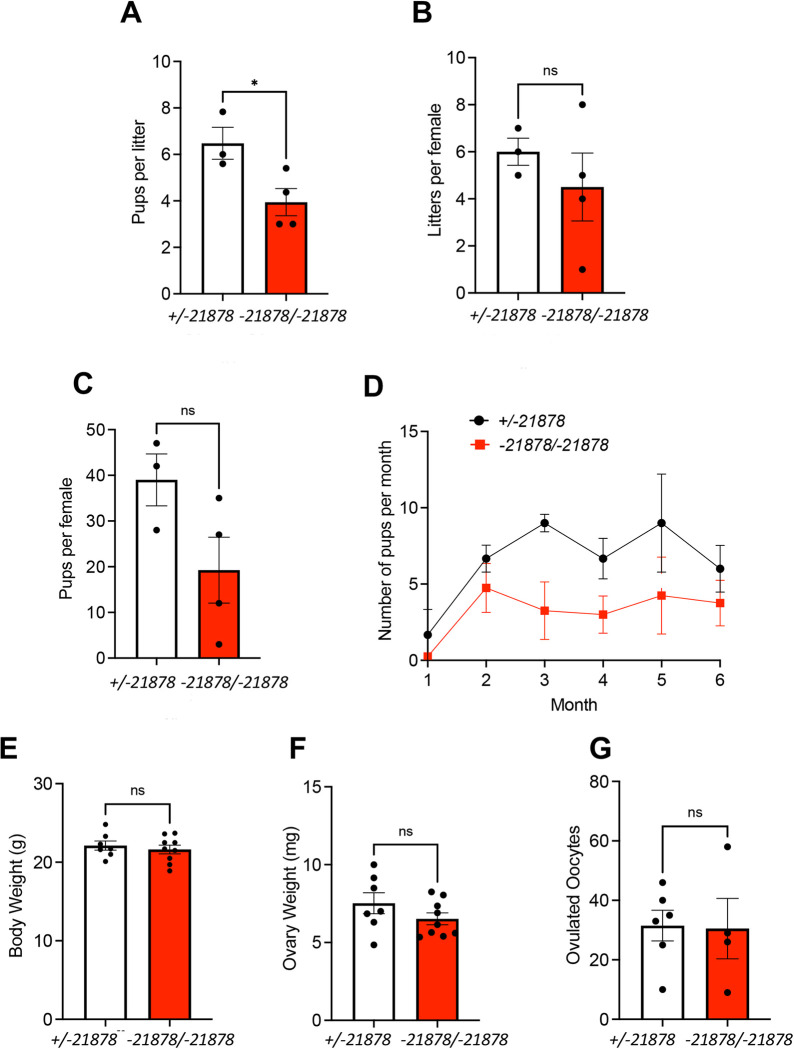
*Wee2* KO causes female subfertility. (A) Average litter size, (B) litters per female, and (C) pups per female of *Wee2* HET and *Wee2*^*-21878/-21878*^ KO female mice mated to WT males over the course of a 6-month fertility trial. Fertility data are also displayed as (D) the average number of pups per month. (E) Body mass average of *Wee2* HET and *Wee2*^*-21878/-21878*^ KO female mice. (F) Average weight of individual ovaries from *Wee2* HET and *Wee2*^*-21878/-21878*^ KO female mice. (G) Average number of ovulated oocytes from *Wee2* HET and *Wee2*^*-21878/-21878*^ KO female mice following superovulation with PMSG and hCG. Data in histograms represent average ± standard error of the mean. Analyzed by an unpaired t-test, * = 0.04.

Homozygous -21878 bp deletion (*Wee2*^-*21878/-21878*^) female mice were pair-housed with one male for 6 months to test their fecundity and the average number of offspring per litter and the number of litters were determined. Relative to *Wee2*^*+/-21878*^ HET females, there was a significant reduction in the number of pups per litter in *Wee2*^*-21878/-21878*^ KO females (HET, 6.50 ± 0.69, n = 3, versus KO, 3.9 ± 0.58, n = 4, p = 0.048) (Fig 2A). There was no difference in the average number of litters per female, the average number of pups per female, or in the number of pups per month (Fig 2B–2D). These data demonstrate that WEE2 has a partial role in the maintenance of fecundity, but WEE2 is not essential for female fertility in mice.

**Fig 3:**
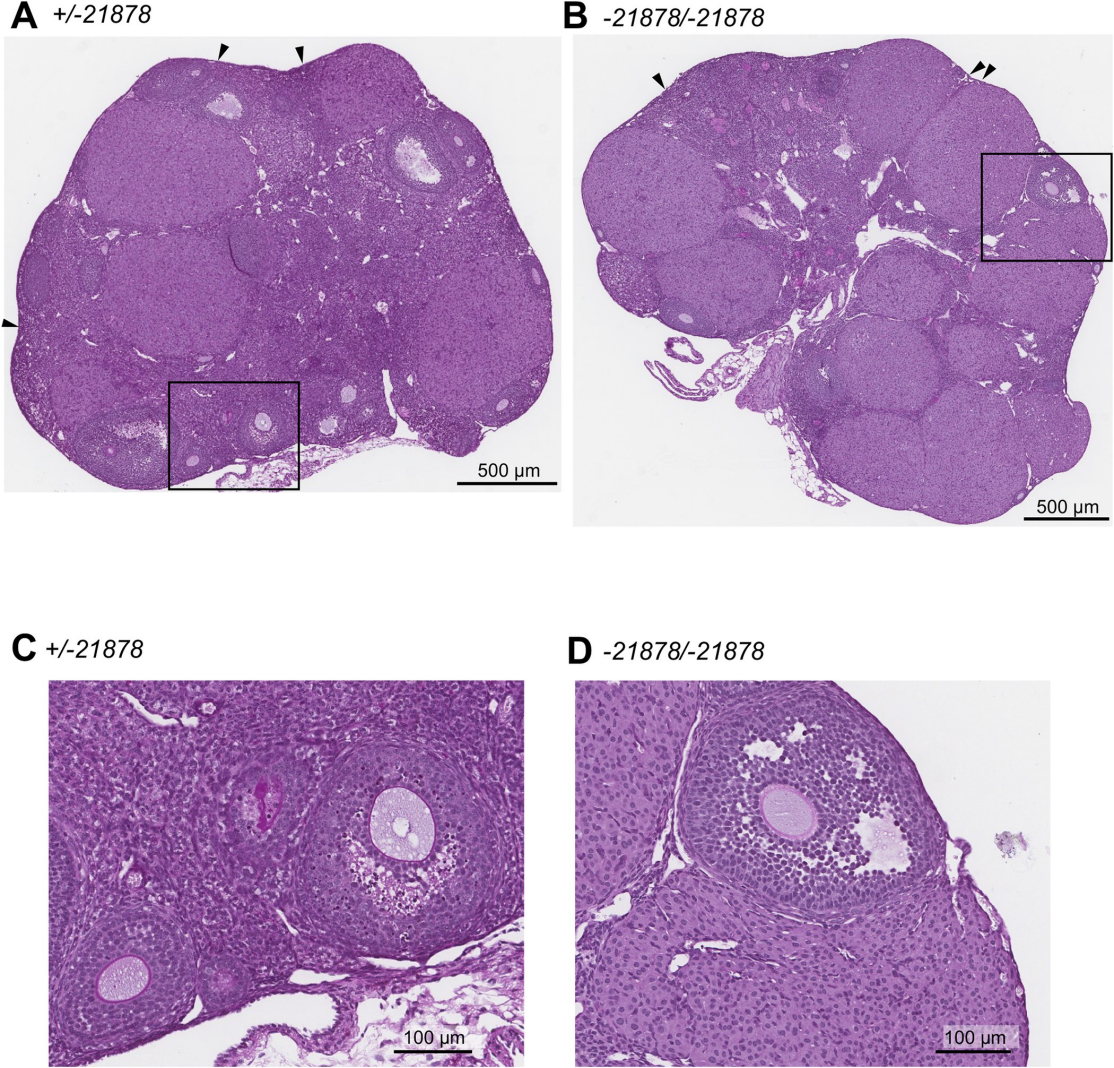
Normal ovarian histology observed in *Wee2* KO mice. (A, B) Representative PAS-Hematoxylin staining of ovary sections from *Wee2*^*+/-21878*^ HET and *Wee2*^*-21878/-21878*^ KO mice. Arrowheads indicate the sites that primordial follicles were observed. (C, D) The representative images of growing follicles from *Wee2* HET and KO, respectively. Squares in Fig 3A and 3B are magnified.

We also analyzed sexually mature *Wee2*^*+/-21878*^ and *Wee2*^*-21878/-21878*^ females in detail. We analyzed five to six-month-old female *Wee2*^*+/-21878*^ and *Wee2*^*-21878/-21878*^ mice and observed no statistical difference in their body weight (HET, 22.1 g ± 0.59, n = 7 versus KO, 21.6 g ± 0.55, n = 9) or ovarian weight (HET, 7.52 mg ± 0.67, n = 7 versus KO, 6.52 mg ± 0.38, n = 9) (Fig 2E and 2F). To determine their capacity to ovulate, we stimulated 3-month-old mice with exogenous gonadotropins and collected oocytes from the oviducts 14 h after hCG and counted the number of oocytes. *Wee2*^*+/-21878*^ and *Wee2*^*-21878/-21878*^ females ovulated a comparable number of oocytes (HET, 31.5 ± 5.17, n = 6 versus KO, 30.5 ± 10.17, n = 4 (Fig 2G). Ovulated oocytes from *Wee2*^*+/-21878*^ and *Wee2*^*-21878/-21878*^ did not show any gross morphological defects using a light microscope (not shown). Histological analysis revealed that ovaries of *Wee2*^*+/-21878*^ and the *Wee2*^*-21878/-21878*^ mice were nearly identical to each other. Both genotypes showed multiple healthy primordial follicles, growing follicles, and corpora lutea (Fig 3A–3D).

**Fig 4:**
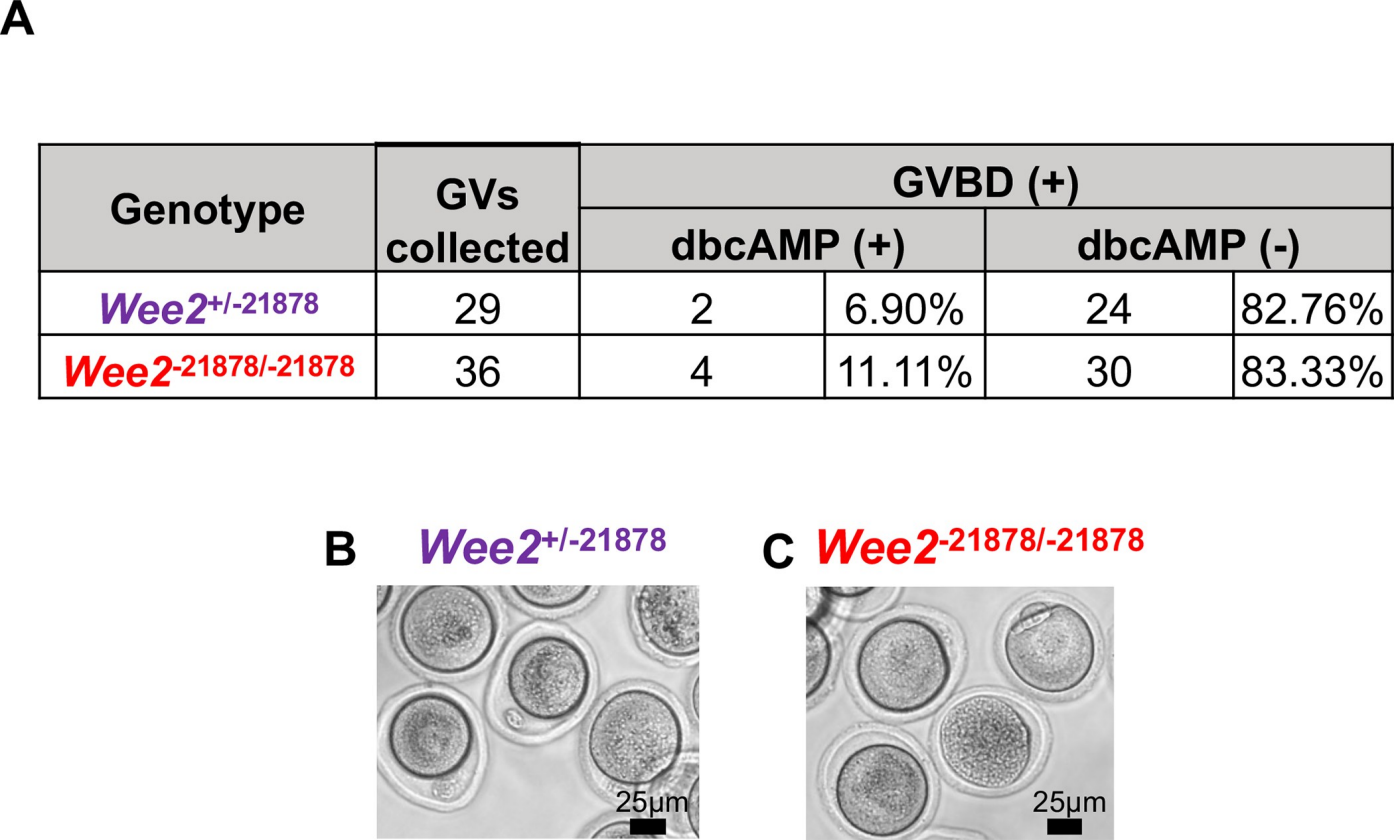
Oocyte maturation *in vitro* is comparable between *Wee2*^*+/-21878*^ HET and *Wee2*^*-21878/-21878*^ KO mice. (A-C) (GV) stage oocytes were collected from *Wee2*^*+/-21878*^ HET (n = 6) and *Wee2*^*-21878/-21878*^ KO mice (n = 6) 46 hours following PMSG stimulation and cultured in medium containing dibutyryl-cAMP (dbcAMP) to maintain high intraoocyte cAMP levels and maintain GV arrest. The occurrence of germinal vesicle breakdown (GVBD) was measured 24 hours after incubation dbcAMP and 24 h after removal of dbcAMP. No differences were observed in the rate of GVBD between the two genotypes when incubated in the presence or absence of dbcAMP. Representative images of oocytes from *Wee2*^*+/-21878*^ HET and *Wee2*^*-21878/-21878*^ KO mice following incubation without dbcAMP.

A prior study in macaques indicated that knockdown of *WEE2* in oocytes using RNA-interference leads to premature meiotic resumption in ~40% of GV-intact oocytes in the presence of a phosphodiesterase inhibitor, which cause high levels of intraoocyte cAMP [5]. Therefore, we analyzed meiotic resumption in the presence of presence of dibutyryl-cAMP (dbcAMP, cAMP analog) [15]. GV stage oocytes were collected from ovaries of PMSG-treated *Wee2*^+/-17^ HET and *Wee2*^-17/-17^ KO mice, then cultured in medium containing 100 μM dbcAMP for 24 hours and the number of GVBD oocytes were counted. After 24h, oocytes were washed and further cultured in medium without dbcAMP for another 24 hours to verify that oocytes could subsequently undergo meiotic resumption. During the first 24 h in medium + dbcAMP, oocytes from both *Wee2*^*+/-17*^ HET and *Wee2*^*-17/-17*^ KO female mice seldomly underwent GVBD. Following dbcAMP washout, *Wee2*^*-17/-17*^ underwent GVBD similar to *Wee2*^*+/-17*^ HET (S1B–S1D Fig). The same experiment was performed in the *Wee2*^+/-21878^ and *Wee2*^-21878/-21878^ KO mice, where we found results that were similar to the *Wee2*^*+/-17*^ HET and *Wee2*^*-17/-17*^ KO studies. Specifically, during the first incubation in dbcAMP, *Wee2*^+/-21878^ and *Wee2*^-21878/-21878^ KO oocytes seldomly underwent GVBD (HET, 6.9% vs. KO, 11.11%), and subsequently progressed to GVBD at equal rates after the dbcAMP washout (HET, 82.76% vs. KO, 83.33%) (Fig 4). We identified that certain oocytes in both genotypes failed to undergo GVBD after the dbcAMP washout and died during the culturing process (HET, 10.35%; KO, 5.56%). Thus, maintenance of GV meiotic arrest and subsequent meiotic progression does not require WEE2 in mice.

**Fig 5:**
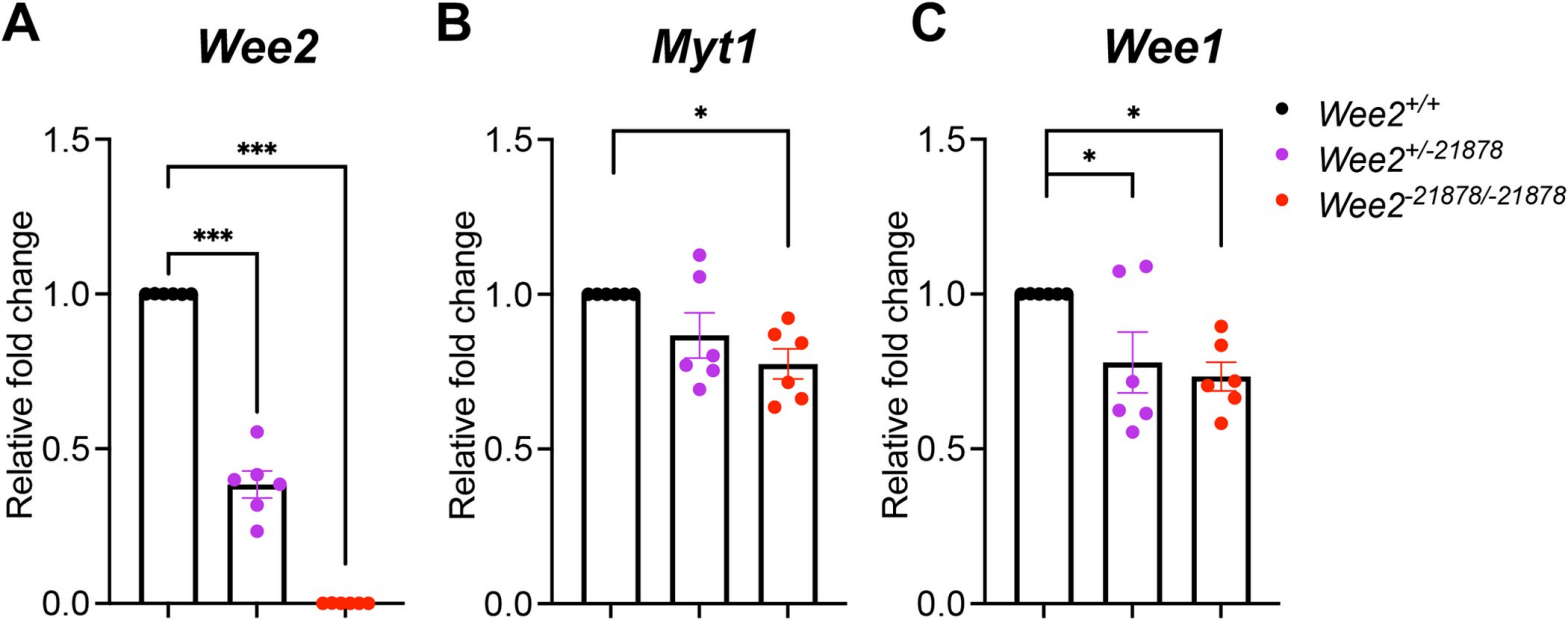
Quantification of WEE-related kinases, *Wee2*, *Myt1 and Wee1*, in the ovaries of *Wee2*^*+/-21878*^ HET and *Wee2*^*21878/-21878*^ mice. (A-C) Gene expression analysis of *Wee2* (A), *Myt1* (B), and *Wee1* (C) transcripts in WT (n = 3), *Wee2*^*+/-21878*^ HETs (n = 3), and W*ee2*^*-21878/-21878*^ KO mouse ovaries (n = 3). *Gapdh* was used as an internal control. Data in histograms represent average fold change ± standard error of the mean. Analyzed by a one-way ANOVA with a Dunnet’s post-test. 0.033 (*), 0.002 (**), <0.001 (***).

One explanation for this finding can be attributed to the presence and activity of additional WEE kinase family members in mouse oocytes [3]. Previous studies showed that other members of the WEE kinase family, such as MYT1, are functionally redundant in the control of GVBD, as the inactivation of both WEE2 and MYT1 was required for GVBD and meiotic resumption [7]. Therefore, we quantified transcript levels of *Wee1* and *Myt1* (*Pkmyt1*) in *Wee2*^-21878/-21878^ KO ovaries (Fig 5). In contrast to *Wee2* expression, which is approximately 50% the levels in heterozygous *Wee2*^*+/*-*21878*^ and absent in the null *Wee2*^-*21878/*-*21878*^ ovaries, both *Wee1* and *Myt1* expression is detected in the *Wee2*^-21878/-21878^ KO oocytes compared to WT or *Wee2*^+/-21878^ HET mice, with a slight but significant decrease in expression in HET and KO mice. Hence, the presence of these additional WEE kinase family members in the oocyte indicates that they could compensate for loss of WEE2.

## Discussion

WEE2 is an oocyte-specific kinase previously proposed as a key factor that regulates meiotic arrest in multiple species [3–5]. Previous studies utilized knockdown and overexpression of *Wee2* in GV oocytes to test its role in meiotic resumption. Models to test the effect of loss of *Wee2* in earlier oocyte developmental stages have not been reported. Therefore, we generated two CRISPR-Cas9 knockout mice to test *Wee2* loss-of-function. We demonstrate that one knockout line shows normal fertility and the second shows a slight reduction in pups per litter, but surprisingly, neither shows gross alterations in oocyte meiotic resumption or progression to MII. Recently, a third *Wee2* KO mouse model was generated by the International Mouse Phenotyping Consortium (IMPC) and their phenotyping pipeline also reports no changes in fertility compared to controls. Thus, these three independent loss-of-function models clearly support that WEE2 is mostly dispensable for overall female fertility in mice [16].

Of our two mutant lines, the female *Wee2*^-21878/-21878^ KO mice produced regularly spaced litters, but show a slight, yet statistically significant, reduction in litter sizes. This minor effect may be a statistical anomaly, as the other two lines do not show this effect. While there was a significant reduction in pups per litter in the *Wee2*^-21878/-21878^ null line, the knockout oocytes were able to undergo meiotic resumption and progression to MII in similar numbers as controls. These data are in line with the previous study in GV mouse oocytes that also report that WEE2 is dispensable for the MI to MII transition [4], most likely due to compensation by the related WEE1 family kinase member, MYT [3]. WEE2 has additional reported activities during fertilization and pre-implantation embryo development [17], which we did not specifically analyze. It is possible that some or part of the subfertility results from defects during these later stages as maternal effect genes can be associated with a spectrum of developmental abnormalities [18], and which will require additional experiments to pinpoint.

Oocyte maturation factors, such as CDC25B and Endogenous Meiotic Inhibitor 2/Early mitotic inhibitor 2 (EMI2) and MYT1 may replace WEE2 and explain the overall lack of phenotype in the three *Wee2* KO mice. CDC25B plays a role in CDK1 activation during the meiotic resumption, leading to MPF activation. CDC25B null female mice are sterile because their oocytes remained arrested at prophase (GV oocytes) with low MPF activity [19]. Cell division cycle 25A (CDC25A) also controls the MI to MII transition by phosphorylating CDK [20]. In *Wee2* KO oocytes, CDC25B may have an earlier role in the initiation of GVBD. EMI2, also known as FBXO43, is downstream and inhibited by CAMKII [2]. EMI2 inhibits the anaphase-promoting complex (APC), establishing and maintaining MII arrest. Reduction of EMI2 causes MII exit, and *Emi2* null male and female mice are sterile due to meiotic defects [21,22]. Activated MPF is inactivated perhaps by inhibiting EMI2, and breakout from the MII phase may be occurring in *Wee2* KO oocytes. These two factors may be more essential than WEE2, while WEE2 might serve as an alternate or ’backup" mechanism to maintain proper meiosis in oocytes and full fecundity.

Several *WEE2* mutations have been identified in humans that result in a variety of WEE2 protein products and failed fertilization in the affected individuals [17]. For example, the study by Sang et al. [10] identified both loss-of-function frameshift and truncating *WEE2* mutations that decreased the levels of WEE2 protein. When expressed in cells, *WEE2* Asp243His missense and *WEE2* Thr493Asn frameshift insertion mutations resulted in decreased WEE2 protein levels relative to the WT WEE2 protein, while *WEE2* His377Tyr frameshift insertion and *WEE2* Glu75Val frameshift insertion mutations resulted in no protein. The corresponding decreased and absent WEE2 protein levels were also observed in oocytes from the affected individuals. Hence, these findings suggest that our WEE2 KO mouse model mimics the *WEE2* mutations observed in humans that result in complete absence of WEE2 protein. However, our mouse model would not fully explain the mechanisms in patients displaying mutations that result in truncated or decreased WEE2 protein levels, given that the physical presence of a defective WEE2 protein might result in a different phenotype due to potential loss of compensation by WEE2-related kinases. Furthermore, while not performed in this study, our mouse model could be used to investigate a potential decline in fertility due to aging. Given that fertility defects could be exacerbated by the presence of *Wee2* mutations in the mice, such longitudinal studies of fertility would be informative and relevant to human health.

Novel non-hormonal female contraceptives are sought to minimize the negative side-effects of currently available hormonal contraceptives. It was reported that women with homozygous *WEE2* mutations experienced pregnancy failure due to fertilization defects [10]. Given this observation and its oocyte-specific expression, WEE2 has been proposed as a potential contraceptive target with lower side effects [11]. Despite the human data and previous reports showing a crucial role for WEE2 in fertilization and fertility in mice, our study and the IMPC KO line showed that *Wee2* KO female mice retained their fertility. Thus, our results indicate that the animal model species used for testing and validation of novel contraceptives targeting WEE2 function needs to be carefully considered in future studies.

In mice for example, the cooperation of MYT1, WEE2, and CDC25 cooperatively regulates MPF activity, while MYT1 does not cooperate in meiotic arrest in porcine oocytes [23]. While we did not observe any compensatory increase in MYT1 expression at the gene level in the *Wee2* KO line, analyses of MYT1 at the protein level could be informative about a compensatory action in these mice. Addressing this question will require studies quantifying oocyte protein levels of MYT1 and WEE1, given that our studies were performed in whole ovaries and both MYT1 and WEE1 are also expressed in somatic cells. In addition, studies at the protein level in oocytes would shed light on the slight but significant decrease in *Myt1* and *Wee1* expression that we observed in *Wee2* HET and *Wee2* KO mice. Given that previous studies have shown redundancy by other WEE-family proteins in oocyte maturation [7], future studies in mice with oocyte-specific inactivation of *Myt1* and/or *Wee1* in the *Wee2* KO mice, would determine their contribution to fertility in the *Wee2* KO mouse lines we generated here.

Accordingly, our results in *Wee2* KO mice suggest uncharacterized inter-species differences in the factors controlling oocyte maturation, where global *Wee2* deletion in mice results in subfertility but may be critical for fertility in humans and primates. Therefore, understanding the contribution of MYT1, WEE2, and CDC25 in meiotic arrest in non-human primates and women will be critical for evaluation of WEE2 as a target for non-hormonal contraception in women.

## Supporting information

S1 Fig Fertility analysis and oocyte maturation *in vitro* in the *Wee2^-17/-17^* KO mice. (A) Average litter size of *Wee2* HET and *Wee2*^-17/-17^ KO female mice mated to WT males during a two-month fertility trial. Litter size was measured by quantifying the total number of pups born. (B) Experimental design of *in vitro* oocyte maturation. The occurrence of GVBD was measured 24 h after incubation with dibutyryl-cAMP (dbcAMP) and 24 h after removal of dbcAMP. (C) The results of occurrence of GVBD. (D) Representative pictures of oocytes from *Wee2* HET and KO after incubation in the medium with/without dbcAMP.
(TIF)

S2 Fig Raw images. **Uncropped gel images for Fig 1B and 1C.** The figures show the full uncropped gel images for Fig 1B upper and lower panels and Fig 1C upper and lower panels.
(PDF)
