# Supplementary figures and images for: Oocyte-specific Wee1-like protein kinase 2 is dispensable for fertility in mice
Source: PLoS One. 2023 Aug 1;18(8):e0289083. doi: 10.1371/journal.pone.0289083 (PMC10393137; doi:10.1371/journal.pone.0289083)

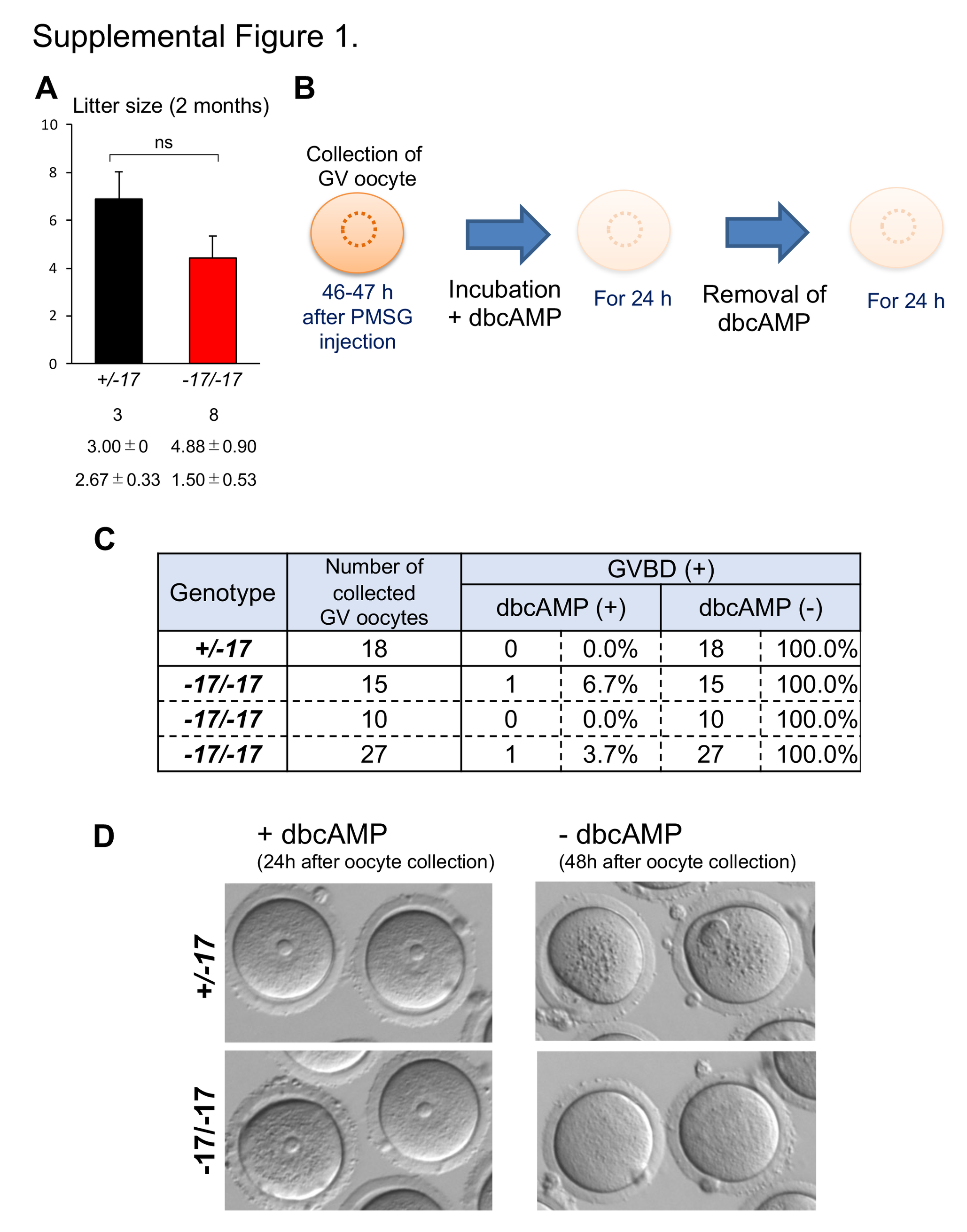

Supplement: S1 Fig — (A) Average litter size of Wee2 HET and Wee2-17/-17 KO female mice mated to WT males during a two-month fertility trial. Litter size was measured by quantifying the total number of pups born. (B) Experimental design of in vitro oocyte maturation. The occurrence of GVBD was measured 24 h after incubation with dibutyryl-cAMP (dbcAMP) and 24 h after removal of dbcAMP. (C) The results of occurrence of GVBD. (D) Representative pictures of oocytes from Wee2 HET and KO after incubation in the medium with/without dbcAMP. (TIF) [file pone.0289083.s001.tif]

Supplemental Figure 2.

Fig1B Upper

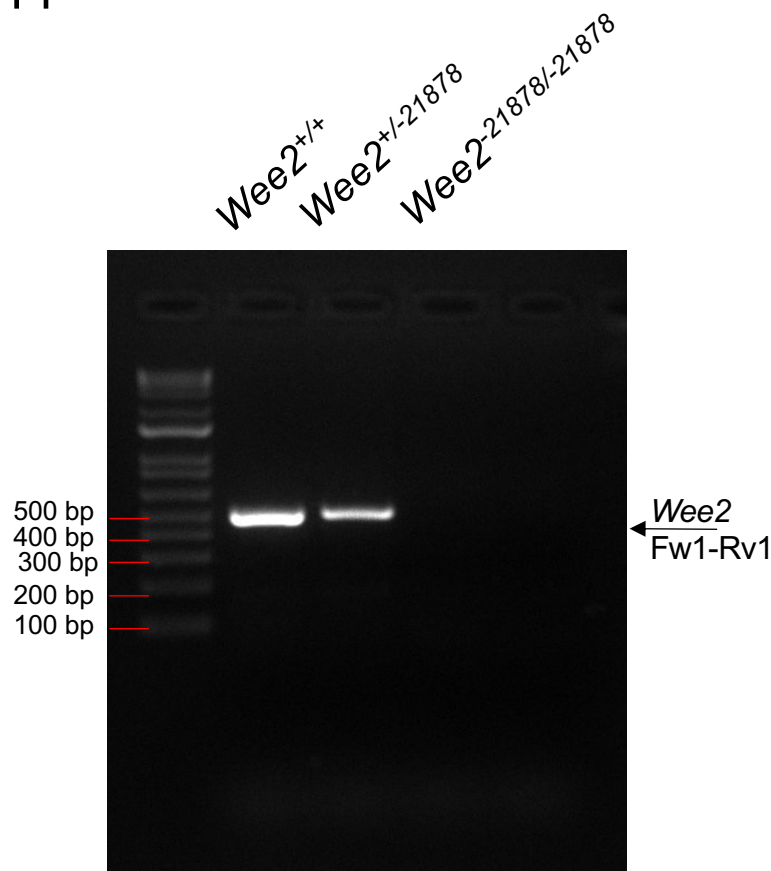

Fig1B Lower

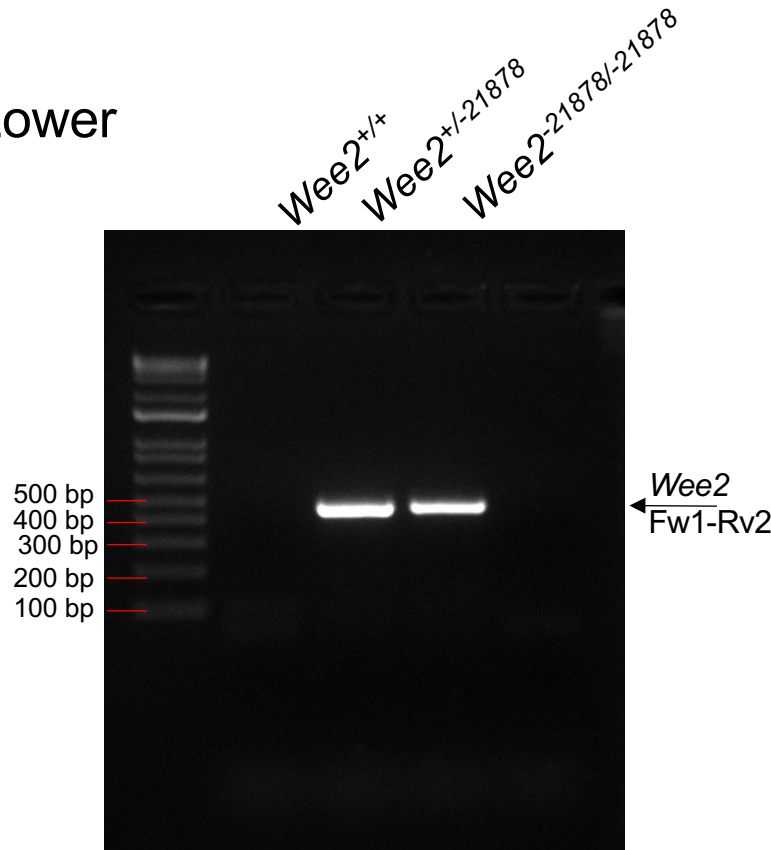

Fig1C Upper

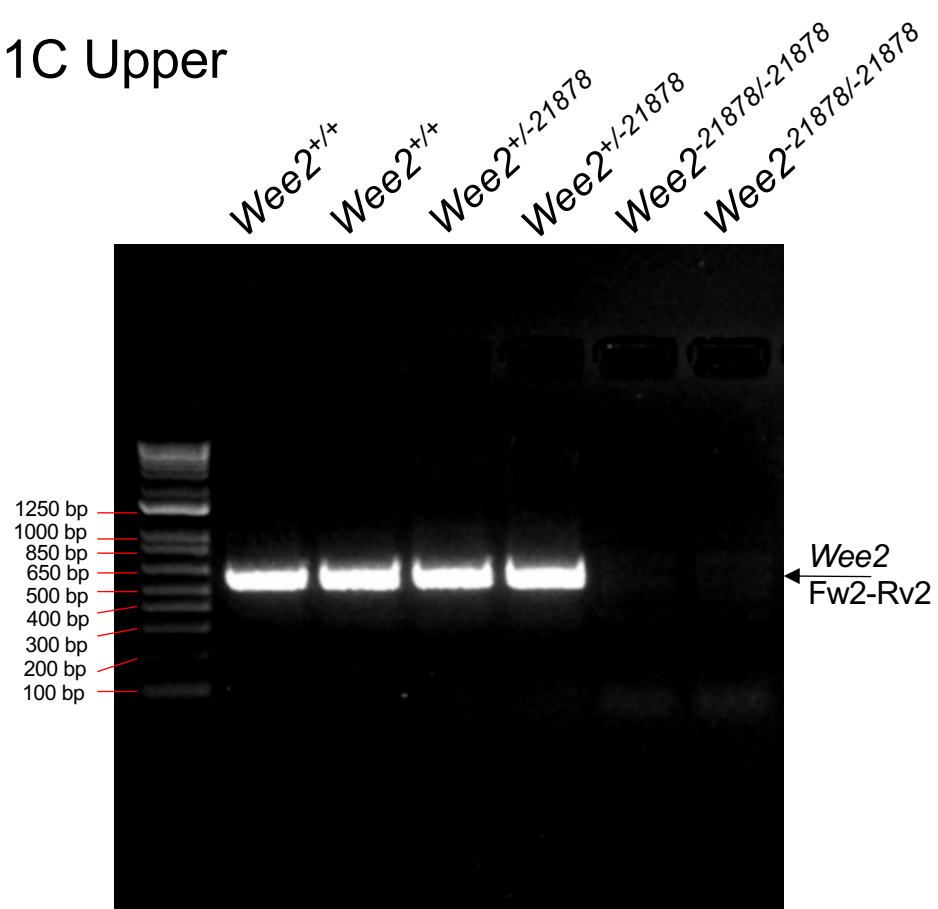

Fig1C Lower

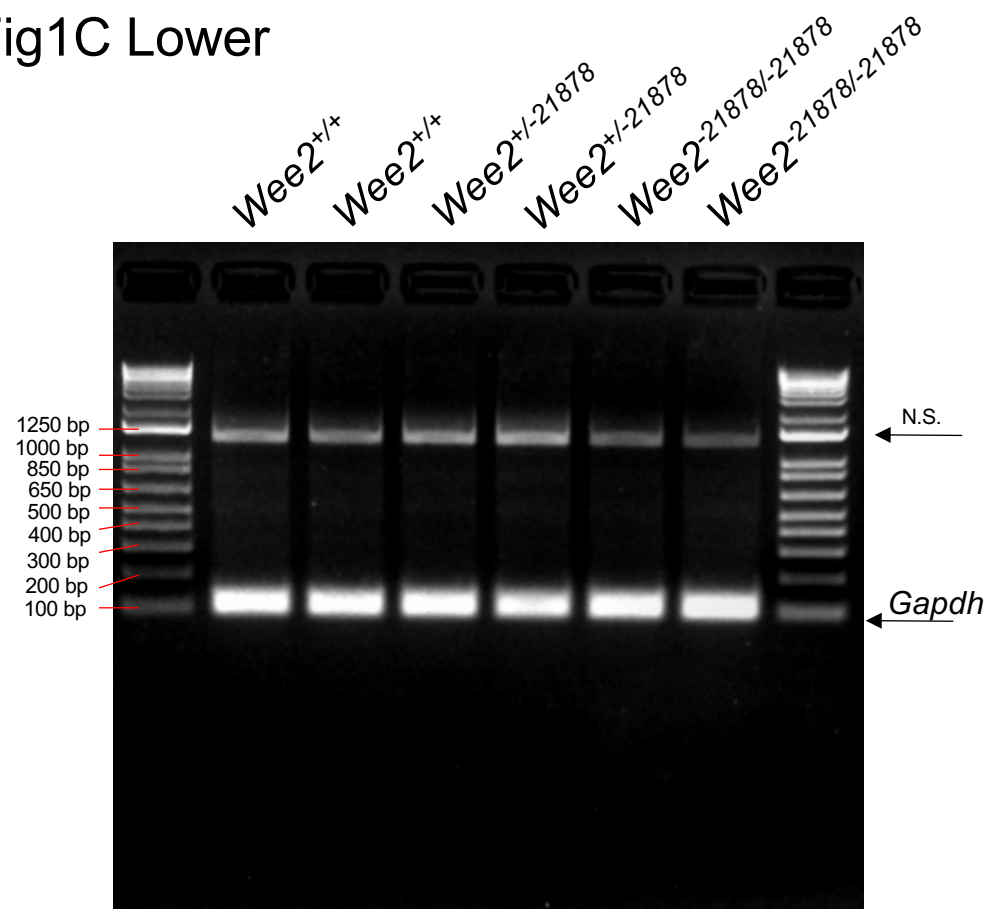

Supplement: S2 Fig — Uncropped gel images for Fig 1B and 1C. The figures show the full uncropped gel images for Fig 1B upper and lower panels and Fig 1C upper and lower panels. (PDF) [file pone.0289083.s002.pdf]
